# Goat Meat: Production and Quality Attributes

**DOI:** 10.3390/foods12163130

**Published:** 2023-08-21

**Authors:** Mariero Gawat, Mike Boland, Jaspreet Singh, Lovedeep Kaur

**Affiliations:** 1 School of Food and Advanced Technology, Massey University, Palmerston North 4442, New Zealand; m.gawat@massey.ac.nz (M.G.); j.x.singh@massey.ac.nz (J.S.); 2 Riddet Institute, Massey University, Palmerston North 4442, New Zealand; m.boland@massey.ac.nz

**Keywords:** goat breeds, carcass characteristics, chevon, capretto, dairy goats, indigenous goat, tenderness, meat composition, meat pH, caprine meat

## Abstract

Goat meat could be a sustainable source of red meat. Its farming requires minimal input, is suitable for free-range farming, and produces a healthier red meat option as it is lean. Although goat meat has advantages for meat production, it still needs to be established as a valuable part of the meat trade market. But, currently, goat meat production is less specialized; there is less intense breed selection for premium meat production, and often the animals are farmed with a multifunctional purpose, such as for their meat, fiber, and milk. The less structured goat meat industry contributes to the inconsistent quality of goat meat. This paper attempts to describe the characteristics of popular goat breeds and indigenous goats as a source of meat and the potential of various goat breeds for meat production. Additionally, this paper presents goat meat’s quality and physicochemical and sensory attributes that are relevant to understanding the unique attributes of goat meat. Much work is needed for the goat meat processing industry to develop its potential.

## 1. Introduction

Meat is an indispensable commodity, highly valued for its nutritional composition. However, the livestock sector was recognised as a significant global warming agent [1,2]. Thus, it is important to alleviate the impacts of the livestock industry by carrying out a more sustainable means of meat production. A goat is considered a multifunctional animal and an untapped meat source with qualities ideal for sustainable red meat production. Goats are highly adaptive to extreme environments, considering production, reproduction, and disease resistance [3,4,5,6]. For example, goats can survive heat stress and prolonged water deprivation [7,8], and in areas with little land space [9]. Goats tend to be more efficient when raised with other ruminants since they are both grazers and browsers, and they eat some grasses and shrubby plants which other ruminants will not [10], rendering them an ideal animal for diversification. Generally, goats depend on the available resources in their environment, and they can be farmed with low inputs (no vitamins or antibiotics). This trait is essential to satisfy the emerging and growing consumer group seeking free-range and antibiotic-free meat for the “lifestyle’’ markets. With the changing environment and evolving consumer preferences, goats will play a vital role as an alternative red meat option, sustaining the meat demand worldwide.

Farming goats also have a downside if not properly managed. Goats can deplete the environment’s natural flora and cause extensive damage to vegetation due to high grazing and browsing pressures. The negative effect of goat meat farming can be mitigated by adequate management requiring knowledge of the varying grazing performances of various breeds since breed type directly affects stocking rates for pasture management [8]. Breeds that match the farming environment can maximize the potential of goat farming since an animal’s capacity to produce the desired product is affected by genetics, environment, and their interaction [11].

Currently, goats are commonly reared without a specific product goal and with less breeding control, specifically for meat production [12]. Thus, compared to the other popular red meats, such as lamb and beef, there is a less formal structure for goat meat production regarding breed selection strategies. This review discusses a brief background of the goat meat industry and the available goat meat products in the market. This paper specifically highlights the significant meat carcass characteristics based on various breeds of goats, grouped according to the purpose for which they are produced, such as for dairy, meat, and fiber, and indigenous and feral goats. This paper also intends to describe goat meat’s quality attributes and how it differs among goat breeds. The data discussed are limited to goat meat, studied without diet manipulation.

## 2. Goat Meat Types, Production, and Trade

### 2.1. Types of Goat Meat in the Market

The export and domestic markets generally classify goat meat into two types, the capretto/cabrito and chevon, differentiated mainly by age and carcass weight [13]. Table 1 presents the description and use of the two main types of goat meat in the market. Cabrito or capretto is the meat from suckling kids popular in various European regions. The cabrito meat is a significant goat meat product in Portugal as part of the country’s culinary tradition and is popular in the Mediterranean lifestyle [14,15]. Together with lamb, goat meat is a popular source of meat for traditional Mediterranean dishes that typically use lean meat. On the other hand, capretto is the term used in Italy, France, many parts of Latin America, and the Caribbean [16,17]. This meat’s distinct flavor and texture make it a premium goat meat product, and its light carcass makes it ideal to be consumed entirely by a family on significant occasions.

Chevon is the popular term for goat meat around the world, and it is the general term used to describe goat meat from adult or mature goats. It is a common meat product traded for export and consumed in various developing countries and a popular choice of meat in India [16,18]. In European countries, most of this meat is used for processed products, which makes it more appealing to a wider range of consumers. Chevon is often discriminated against capretto/cabrito since fresh suckling-kid meat is regarded to have higher edible quality [14]. Moreover, chevon is also known to be a tough meat characterized by its “goaty aroma/sweaty aroma” [19], a flavor that resembles that of mutton or lamb [20].

**Table 1:** Goat meat types, their description, and their use.

Types of Meat	Description	Uses
Capretto/Cabrito	Meat from milk-fed kids, up to 12 weeks, and a carcass weight of 4–12 kg. The meat is described to be light/pale in color and has a fine texture, tender, and lean.	Roasted like a lamb or, in the form of chops, it may be broiled or fried.
Chevon	Meat from young (1–2 years old) to adult goat (2–6 years old). Typical carcass weight ranges from 14–22 kg.	In curries, braises and stews, it requires moist, long, slow-cooking methods.

Sources: [14,15,16,21,22].

To facilitate goat meat trade, an international language between buyer and seller was also introduced by the United Nations Economic Commission for Europe (UNECE) and published in 2007 [23]. In this code system, goat meat is generally referred to as caprine meat and was assigned to Species Code 50. Table 2 shows the adapted category for goat meat from UNECE. In this classification, the sex of the animal is particularly considered.

### 2.2. Goat Meat Production and Trade

Goat meat is a significant source of red meat that still needs to establish itself as a valuable player in the meat export market. In the global market, goat meat is traded as chilled meat from the meat of adult goats. It is mainly produced in developing regions such as Asia and Africa, with less organized farming systems. But these regions have a low contribution to goat meat export since goat meat production is mainly for domestic consumption [24,25]. In developed countries, goat meat is considered exotic, commonly consumed mainly by migrants, and not widely farmed [13]. It is also a preferred meat in Muslim countries, influenced by religious beliefs. In fact, the top destinations for goat meat exports next to the U.S.A. are developed Muslim counties such as the United Arab Emirates, Saudi Arabia, and Qatar (Table 3). Furthermore, China and its provinces also import a lot of goat meat.

In goat meat export, Australia is leading, with approximately 44% average share of the total world goat meat exports, in the past 10 years. Australia is followed by the African countries Ethiopia (22%) and Kenya (7%) (Table 4). Furthermore, goat meat farming is an important agricultural sector in Mediterranean countries. Spain and France also significantly contribute to the production of goat meat for export. In previous years, there has been a significant increase in the production of goat meat as well as sheep and cattle, attributed to the recent problems in the pig industry causing a shift in demand for other meat options [26].

## 3. Goat Meat from Various Breeds

Goats are sold based on their live weight and its relationship to their carcass yield [27]. The carcass characteristic determines the meat quality and value of the animal before it reaches the consumer. Among ruminants, goat carcass has been described to contain proportionately less fat with more carcass muscle and bone than sheep [4,21,28,29]. A breed that can produce significant carcass yield and high saleable meat is more valuable. As with other animals, the distribution of tissues in goats is relative to maturity, sex, breed, age, and nutrition [30]. Goats’ phenotypic characteristics and production potential vary greatly, affecting carcass and meat quality. Research shows contrasting reports on dressing out percentage values among breeds [21,31,32]. However, when different goat breeds have the same frame size (small vs. large framed goats), their carcass’ quality is often highly influenced by their size more than by breed type [13,33]. This section attempts to describe the characteristics of popular goat breeds as a source of meat that is categorized according to the main purpose for which they are produced, such as for meat, fiber, and dairy.

### 3.1. Meat Breeds

Meat breed animals are described as having large body sizes for a higher carcass yield. Additionally, meat producers require low-fat and easily de-haired animals [16]. Among goat breeds, some known meat breeds are the South African Boer or Boer, Spanish, Kalahari, and Savannah. Boer is the most popular among the meat breed goats, and it is identified as having the size, body weight, bone structure, and growth rate needed to fulfill all the market requirements [10,34,35]. Boer has a high dressing percentage that ranges from 50–57% [16] and tends to have a higher fat cover than most meat-breed goats [36]. Compared to Spanish goats, Boer have higher body weight, carcass weight, fat thickness, and a high proportion of muscle, indicating desirable carcass quality [32]. Boer have genetic information linked to size and muscularity close to cattle and sheep [37].

Spanish goats, a popular meat breed in the United States of America (U.S.A.), have less fat thickness and a dressing percentage of around 51–53.2% [38,39]. The Kalahari and Savannah breeds resemble Boer; Kalahari is described as a handsome breed that looks like Boer in form but is red with a slightly lighter body weight [40]. Savannah breed, from the native goats of Southern Africa, is all white, with a medium to large body frame, and its mature weight is about 60 kg [16]. Other known meat breeds in some countries are Kiko and Kikonui, the two purpose-bred meat goats in New Zealand, with Kikonui being an improved Kiko [41]. Although Kiko is not as popular as Boer in that country, it has been a successful breeding stock in the United States. It is an improver breed that can add size, growth rate, and milk production to local stock without reducing hardiness. Solaiman et al. [42] reported that a Kiko kid was comparable to a Boer kid regarding carcass characteristics. Other meat breeds superior for goat meat production are the Galla in East Africa [43] and the Katjang in South East Asia [4].

### 3.2. Dairy Breeds

Dairy breed goats comprise a large portion of the goat meat market since culled dairy goats are sold as meat [44]. Dairy goats have the advantage of large body sizes suitable for meat production, with a dressing percentage that averages 50%. Additionally, these breeds can have multiple births [16]. The popular dairy breeds are Saanen, British Saanen, Sable, Alpine, and Anglo-Nubian. Saanen was reported as the most widely distributed breed in the world [8]. Regarding meat production, Anglo-Nubian, a dual-purpose breed for meat and milk production, was reported to have better carcass characteristics than British Saanen and has a large proportion of meat and lower fat content [45]. Alpine goat is also a highly developed dairy goat, recognized for its size, being ideal for meat production, and its high milk yield. Since milk production is the primary purpose of dairy breeds, goat meat from dairy goats is often from culled goats of ages 5–6 years, producing significantly tough meat that is often discriminated against by consumers [27]. The toughness of the meat is related to a higher mechanical and chemical stability of collagen fibrils as the animal ages [46]. Since meat from culled dairy goats is abundant, its toughness is often generalized to the quality of goat meat in the market.

### 3.3. Fiber Breeds

Goats are also farmed as a source of fiber. The known fiber breeds are Cashmere, Angora, and Cashgora. In general, the fiber breeds are not ideal for a high-yielding goat in the market in terms of its carcass and meat quality. For example, Angora goats are unsuitable for meat production since they have been intensely bred for higher mohair production while body weight has been compromised [12]. Because of its small size, it only has an average carcass weight of 13 kg [4,47]. Additionally, the Angora goat is high in fat, low in lean content in major cuts [47], and has been described as having a stringy texture [16]. Cashmere kids, another fiber goat, were reported to have a significantly lower rating for meatiness than crossbred (Cashmere × (Boer × German Fawn)) [48]. Generally, fiber-breed goats have been consistently found to have high fat content when crossed with other breeds [36,47].

### 3.4. Indigenous or Native Breeds

Indigenous breeds or native breeds are not popular compared to commonly imported breeds. However, this local livestock is becoming in demand because of its adaptability. Widely studied indigenous goats are found in South Africa where the goat is the most valuable ruminant in the region. The size and dressing percentages of South African indigenous goats can be similar to Boer [32,49], and are considered to have a high potential for chevon production with a reported dressing percentage that ranges from 38–55% [50]. Veld goats, for instance, have a comparable total meat and total bone percentage to that of Boer [49]. However, the meat output of indigenous goats can also differ from Boer due to varying proportions of bone-to-muscle content. South African indigenous goats tend to have high bone content attributed to poor muscling [32].

The pattern of high dressing meat percentages for indigenous goats is relative to their size. For example, large-framed indigenous Iranian goats had higher dressing percentages than their lighter counterparts [51]. The Jebel Akhdar breed, which are large-framed goats, are considered to have a high potential for goat meat production because of their size [52]. Some other reported dressing percentages for indigenous goats were 56.5% for native Omani goats [53], 47% for West African dwarf goats [54], and 38–50% for South African indigenous goats [50].

In Europe, various conservation and development programs are under way for the potential of native breeds in goat meat production. Some of the currently conserved native or indigenous breeds are Cilentana [55] and Teramana goats of Italy [56], and Carpathian goats of Poland [57].

Generally, there is a wide range of variation in body weight characteristics of indigenous goats, and there is high potential for the improvement of the breed. The high adaptability of these groups is crucial in meat production since fitness or adaptive traits have high importance for tropical livestock production [53].

### 3.5. Feral Goats

Feral goats are the known wild goats from the dairy or fiber breed goats that escaped or were freed and established their habitat in islands and mountainous areas [58]. They are abundant in New Zealand and Australia and play a significant role as a major source of meat for export because of their availability. The average carcass weight of a feral goat was reported to be 10 kg, generally lower than goats usually killed for export [59]. Compared to dairy goats, feral goats are smaller [60]. Male feral goats were heavier than females, and the dressing percentage for the same average weight at slaughter was higher for males (44.6%) compared to females (42.2%) [59]. Australian and New Zealand feral goats yield lower carcass percentages of 43.4 and about 44.6%, respectively, compared to the carcass yield of meat breed goats that reaches up to 50% [59,60]. Overall, the feral goat was described as producing very lean meat with a low carcass yield. The smaller carcasses of feral goats suggest an undesirable trait for meat production. However, despite a low meat yield from these breeds, their population contributes significantly to goat meat production. Australia is the largest goat meat exporter in the world, and it exploits its feral goat population to produce goat meat.

## 4. Meat Quality

Meat quality is a set of intrinsic and extrinsic characteristics related to a consumer’s perception or expectation during purchasing, consumption, and processing [46,61,62]. The inherent characteristics, such as texture and flavor, can be sensed while eating [63]. On the other hand, extrinsic traits are associated with how the meat is produced, focusing on animal welfare and ecological impact [46]. Overall, meat quality is a multidimensional concept that includes key quality dimensions such as sensory, nutrition, safety, and convenience and the story behind how the meat is produced. For this review, goat meat quality will be discussed in terms of the most significant quality parameters in food processing technology: chemical composition and physicochemical and functional properties.

### 4.1. Chemical Composition

Skeletal muscles contain 75% water, 20% protein, 1–10% fat, 1.2% carbohydrate, 0.65% minerals, and <0.1% vitamins [64,65]. Generally, the proximate composition of goat meat was significantly affected by genotype [13,38,66,67]. Among the popular meat sources, goat meat is a source of lean meat with ~18–25% protein content for raw meat [32,59,68]. Goat meat was identified as having a favorable protein-to-fat ratio due to lower intramuscular fat (IMF) content per gram of meat [32].

The fat content of goat meat is comparable to poultry meat and significantly less than beef and lamb [20,33,69,70]. Although in a study by Tshabalala et al. [32], Boer goat contained a comparable amount of fat to lamb, and this is consistent with various studies [10,42], confirming that among goat breeds, Boer tends to have high fat content (Table 5). Aside from Boer, known dairy breed goats had the highest fat content. On the other hand, the feral goat was identified to be a very lean meat with fat content ranging from 0.9 to 1.5% [60,71,72]. Goats having very low IMF (below 2%) could be related to the maturation rate in combination with their grass diet. As frequently reported, beef from late-maturing breeds and raised on pastures had lower IMF [73]. Moreover, goats develop poor subcutaneous fat at a slow rate [30,74] which can also be explained by the adaptability of goats in the tropics, where they tend to deposit more fat in the viscera rather than in the subcutaneous region [75,76].

The fat composition of ruminants also determines the quality of meat. For example, the ratio between polyunsaturated fatty acids (PUFA) and saturated fatty acids (SFA) is an index used to measure the impact of a specific fat source on cardiovascular health [77]. A higher PUFA/SFA value means a healthier fat source. The reported PUFA/SFA in goat meat is low (0.16–49) and close to that of lamb (0.10–0.37) [66,78]. However, it is significantly lower than PUFA/SFA in fish (1.12). Young goats’ fatty acid (FA) profile is related to the composition of the milk they consume. In contrast, the FA profile for adults is relative to the activity of rumen bacteria in response to their diet [79]. Additionally, FA profiles can be altered with varying energy levels in the animal’s diet [80].

Like cattle and sheep, goats produce meat with high saturated fat content because ruminants have unique rumen bacteria capable of the biohydrogenation process of converting unsaturated fatty acid to saturated fatty acids [81]. Goat fat mainly comprises oleic, palmitic, and stearic acids [32,67,78,82,83,84]. Goat fat contains higher amounts of polyunsaturated fatty acids (PUFA) than lamb and beef, and it is mainly linoleic (C18:2), linolenic (C18:3), and arachidonic (C20:4) [66,78].

In a comprehensive review by Banskalieva et al. [78], breed and muscle type significantly affect goat meat’s fatty acid profile. Boer goats, for example, have been reported to contain higher unsaturated fatty acids (UFA), MUFA, and PUFA compared to feral goats [32]. 

**Table 5:** Reported proximate composition of raw goat meat from various breeds.

Breeds	Breed	Age and Sex	Muscle Type	Moisture (%) (Wet Basis)	Protein (%)	Fat (%)	Ash (%)	Source
Meat breeds	African Boer	Castrated males without any permanent incisors	Soft tissue	69.4	22.8	10.5	0.95	Tshabalala et al. [32]
	Boer	Intact and castrated male, 8–10 m	Loin	75.1–76.3	20.0–20.3	2–2.8	0.9–1.0	Gawat et al. [72]
Dairy breeds	Alpine	Four years	*Gluteus superficialis*	74.6	19.52	3.9	1.0	Ivanović et al. [85]
	Saanen	Four years	*Gluteus superficialis*	74.8	19.82	4.1	1.0	Ivanović et al. [85]
	Guanzhong dairy breed	6–10 m (sex, unspecified)	*Longissimus thoracis*	75.5	19.58	4.82	1.0	Ding et al. [86]
Indigenous	Balkan goat	Four years old, female	*Longissimus dorsi*	74.5	20.6	3.9	1.0	Ivanović et al. [87]
	Serbian white goat	Four years old, female	*Longissimus dorsi*	75.4	20.0	3.6	1.1	Ivanović et al. [87]
	East African Tanzania indigenous goats	1.5–2 years old, male and female	*Longissimus thoracis et lumborum*	70.6	23.45	2.5	4.4	Shija et al. [88]
	Sarda goats	Kids (42 days) male and female	*Longissimus dorsi*	75.6–75.9	21.9–22.2	1.0	1.2	Vacca et al. [89]
	Indigenous Veld goat	Male and female, 9–12 m	Loin	75.6–76.8	19.6–20.1	1.6–2.7	1.0–1.1	Van Wyk et al. [90]
Feral goats	Australian feral goat	Unspecified age, slaughter weight 5–70 kg	*Longissimus thoracis*	75.9	20.7	1.25	1.1	Werdi Pratiwi et al. [60]
	New Zealand feral goat	12–24 m	*Longissimus thoracis*	71.7	23.1	0.9	1.16	Gawat et al. [72]

### 4.2. Physicochecmical Properties

#### 4.2.1. Meat pH

The inherent ultimate pH (pHu) is an important quality determinant influencing meat’s processing and sensory attributes [91,92]. The meat’s ultimate pH level can be grouped into three: high (pH > 6.3), intermediate (pH > 5.7 to pH < 6.3), and low (pH < 5.7) [92]. The typical ultimate pH for red meat ranges from 5.4–5.9 [93]. Goat meat, on the other hand, is extensively reported to have high pH, and its reported optimal pH ranges from 5.5 to 6.2 (Table 6). The high pH of goat meat has been attributed to its tendency to be prone to perimortem stress [68]. This has been supported by the perimortem concentrations of glycolytic metabolites in goat muscles with low glycogen concentration [16,94,95]. The normal pH of goat meat (intermediate pH, ~5.7–6.3) plays a significant role in the bio chemical processing taking place post-mortem. Goat’s intermediate pH can maintain the stability of lysosomes throughout the aging process [96], thus limiting lysosomal enzyme activity; this can explain why goat meat is unresponsive to aging for meat tenderization.

#### 4.2.2. Water-Holding Capacity (WHC) and Cooking Loss

Water-holding capacity (WHC) is the property of meat to retain its naturally occurring water when applied with external force. It is primarily influenced by the ability of the myofibrillar system to immobilize tissue water [27]. Goat meat was reported to have superior water-holding capacity and less cooking loss compared to lamb [69,70,76,100] and poultry [70], which can be explained by the influence of meat pH on WHC. As the pH declines, proteins will have a reduced capacity to retain water and a subsequent increase in water intracellularly [101,102]. Thus, higher pH meat such as goat will have significantly higher WHC.

Reports on the ability of goat meat to retain moisture after cooking vary, ranging from 10–28% cooking loss [14,76,88]. The reported cooking loss for goat meat breeds is presented in Table 7. Goat meat appears to have a lower cooking loss than sheep [69,88], although some studies do not agree [76,80,103]. Between goat breeds, differences in cooking loss were inconclusive. For example, sous vide-cooked New Zealand feral and cross-bred Boer showed no differences in cooking loss [72], but differences in cooking loss were observed in other studies [14,21]. The observed cooking loss values for goat meat are highly affected by muscle pH [104].

#### 4.2.3. Collagen Content and Solubility

Proteins from meat are also rich in collagen, the main composition of muscle intramuscular connective tissue (MICT), which plays a significant role in the “background toughness” of meat [105]. The amount and the extent to which collagen crosslinks depends on muscle type, species, genotype, age, sex, and level of physical exercise [102]. In general, the higher the insoluble collagen content, the tougher the meat [32]. Goat meat was reported to have lower collagen solubility when compared to mutton [32,106,107] and beef [108]. Goat meat would highly benefit from a process that can gelatinize collagen during cooking or the application of a process or enzyme that targets significant structural weakening of connective tissues.

When it comes to meat toughness, both the collagen content and its solubility are vital. Goat meat has been reported to contain significantly higher collagen levels than sheep meat [106,109]. The high collagen content in goat muscles can be explained by how goats forage for food, where their muscles get used more, leading to more developed tissue. It was reported that between feral and Boer goats, feral had higher collagen content, explained by how feral goats grazed on hilly slopes [72]. The same observation was also reported for mountain goats (white Carpathian goats in Poland), where mountain goats generally have higher collagen content compared to the meat of the Saanen goats [57].

**Table 7:** Cooking loss of goat meat from various goat breeds after 24 h post-mortem at 4 °C storage temperature and cooked at 60–80 °C internal temperature.

Species or Breed	Muscle Type	Cooking Method	Cooking Loss (%)	Source
Serrana (S), Bravia (B), and Serrana Bravia (SB) crossbred, (male and female kids)	*Longissimus thoracis et lumborum*	Sealed plastic bag in a water bath at 80 °C for 1 h	10.2–12.2	Shija et al. [88]
Nebrodi goats (male and female, 47 days old)	*Longissimus dorsi*	Sealed plastic bag, cooked until the internal temperatures reached 75 °C	14.9–16.5	Todaro et al. [66]
Tanzania indigenous goats (5–2 years old)	*Longissimus lumborum*	Sealed plastic bag in a water bath at 80 °C for 1 h	18.8	Shija et al. [88]
Yearling Indian goats	*Longissimus dorsi*	1 h in a water bath at 80 °C (packaging not specified)	22.7	Sen et al. [76]
Omani goats	*Longissimus dorsi*	Water bath at 70 °C for 90 min (packaging not specified)	24.0	Kadim et al. [110]
Saanen × Thai native	Leg	Sealed plastic bag and cooked in a water bath at 80 °C for 10 min	27.4	Wattanachant et al. [111]
Anglonubian × Thai native	Leg	Sealed plastic bag in a water bath at 80 °C for 10 min	27.8	Wattanachant et al. [111]

### 4.3. Sensory Qualities

#### 4.3.1. Meat Color

Goat meat tends to be darker meat than other red meat [106,112,113], which can be explained by the high pH of goat meat and low IMF. The effect of genotype on the color properties of goat meat is still debatable. Some authors reported no observed differences in meat color between Kiko and Boer [42,114] and among Batina, Dhofari, and Jabal Akdha breeds [97]. At the same time, many studies have also presented the effects of breed on meat color among breeds. Dhanda et al. [21] and Santos et al. [14] reported that genotype significantly influenced goat muscle color. Gawat et al. [72] and Peña et al. [67] reported that genotype generally significantly affects redness values. The difference in redness values between genotypes reported in literature often correlates to the amount of heme pigment in a muscle [14,21,36]. Muscle pigment concentration is directly influenced by many factors, such as species, age, type of muscle, and physical activity.

#### 4.3.2. Shear Force Values

The instrumental measurement of tenderness is expressed in peak shear force (PSF) in N or Kg; the higher the shear force values, the tougher the meat. Although there is a high correlation in using shear force for describing the toughness of meat, direct comparisons between reported values in literature are difficult due to the differences in the methods used. Table 8 shows some reported values for the instrumental tenderness of goat meat, assessed using Warner-Bratzler shear force (WBSF). PSF values of 40 N or 4.1 Kg will be used as the threshold for acceptable tenderness [115].

Goat is known to exhibit tough meat and is generally regarded as inferior to other red meat regarding tenderness perception. On industrial scale, electrical stimulation is used to accelerate post-slaughter biochemical changes of goat meat to improve its texture [113]. It is widely reported that goat meat had WBSF values higher than 5 Kg. High shear force values of goat meat have been linked to high collagen content, low collagen solubility, muscle fiber characteristics, and low intramuscular fat [88]. Kadim and Maghoub [27] explained that goat meat is tough because most of the goat meat sold is from slaughtered adult goats with decreased collagen solubility. In addition, a goat’s lack of a thick subcutaneous fat layer predisposes it to cold shortening during rapid chilling [72,95,104], which happens due to a small goat carcass, with less fat cover, facilitating a fast decrease in temperature that induces cold shortening [68,72,104]. Furthermore, the inherent high pH of goat meat also can hinder the endogenous proteolytic enzymes that could exert a tenderizing effect during aging.

Upon examining the breed type, differences are apparent if animals compared are from different groups, such as dairy breed vs. fiber breed vs. meat breed. However, the effect of genotype on goat meat tenderness is not clear, and it is also because meat tenderness is a complex trait resulting from multiple factors that involve pre- and post-mortem handling, genetics, and rearing conditions. Boer goats, having elevated levels of IMF compared to other goat breeds, have an advantage when it comes to having more tender meat due to the effect of IMF on the perceived texture of the meat.

## 5. Conclusions

Goat farming has many advantages for the future of the red meat industry, but its value in the red meat sector is not as established in Western markets as beef or lamb. Goats are multi-purpose animals, but when it comes to meat production, the larger the dressing percentage, the higher the marketable value. Hence, goat meat production requires a breed that produces good carcass quality. But the challenge is maximizing goats’ high adaptability, considering the breed and the environment. This condition makes it ideal to exploit the potential of indigenous goats for meat production. Regarding meat quality, goat meat is known to be a source of lean meat. However, its toughness is an issue that can be attributed to the combined factors of high pH, high collagen content, and less fat content. Additionally, because of the less structured goat meat production, there is less quality control for goat meat sold in the market; meat from culled dairy goats often contributes to the image of goat meat as tough meat. The toughness of goat meat should be addressed using innovative processing technologies suitable for its characteristics. Moreover, because of the unstructured nature of the goat meat industry, better-managed processing in an abattoir can improve goat meat quality. For example, a dedicated cold storage condition tailored for goat meat carcasses can address cold shortening issues. A lot of work is still needed to develop the goat meat industry’s potential.

## Figures and Tables

**Table 2:** Categories of caprine meat (adapted from the United Nations Economic Commission for Europe (UNECE)) [23].

Caprine Category Code	Category	Description
1	Kid (Chevreau)	A young Caprine under 3 months of age, which does not have any permanent incisor teeth.
2	Kid	Caprine under 6 months of age which does not have any permanent incisor teeth.
3	Wether (Capra)	A young castrated male Caprine having one but not more than two permanent incisor teeth.
4	Nanny (Doe)	A mature female Caprine having more than one permanent incisor teeth.
5	Billy (Buck)	A mature male Caprine having one or more permanent incisor teeth.
6	Goat	Any Caprine animal—male and female

**Table 3:** The top goat meat (fresh or chilled) importing countries in the past 10 years (2011–2021) [26].

Country	Average Value (Tonnes)	% of the Total Volume
United States of America (U.S.A.)	17,387.13	28
United Arab Emirates (U.A.E.)	13,163.40	21
China	5630.54	9
Saudi Arabia	3652.07	6
Qatar	3343.52	5
China, Taiwan Province of	3261.14	5
Oman	2707.72	4
Canada	1750.69	3
Portugal	1489.11	2
China, Hong Kong S.A.R.	1439.02	2
Other countries	8553.72	14

**Table 4:** The top goat meat (fresh or chilled) exporting countries in the past 10 years (2011–2021) [26].

Country	Average Value (Tonnes)	% of the Total Volume
Australia	26,818.61	44
Ethiopia	13,797.09	22
Kenya	4143.68	7
France	2459.98	4
Pakistan	2416.82	4
China	2212.84	4
China, mainland	2160.47	4
Spain	2056.20	3
New Zealand	1183.23	2
Jordan	884.40	1
Other countries	3424.81	6

**Table 6:** Meat pH after 24 h chilling.

Breed	Age and Sex	Muscle Type	Ultimate pH	Source
Omani goats	One-year-old male	*Longissimus dorsi*	5.6–5.8	Kadim et al. [97]
Boer	Kids to adult	*Longissimus*	5.7–6.0	Werdi Pratiwi et al. [35]
Anglo-Nubian	Male kids	*Longissimus thoracis et lumborum*	5.7	Peña et al. [67]
Serbian white goat (domestic goat)	Four years	*Longissimus dorsi*	5.7	Ivanovic et al. [87]
Balkan goat (domestic goat)	Four years	*Longissimus dorsi*	5.7	Ivanovic et al. [87]
Indigenous Veld goats	9–12 m intact and castrated	*Longissimus thoracis et lumborum*	5.7	Van Wyk et al. [90]
Criollo Cordobes	Male kids	*Longissimus thoracis et lumborum*	5.7–5.8	Peña et al. [67]
Serrana, Bravia, and Serrana X Bravia crossbreeds	kids, male and females	*Longissimus thoracis et lumborum*	5.8	Santos et al. [14]
Alpine	4–8 m	*Longissimus lumborum*	5.8–5.9	Saccà et al. [98]
Indigenous Serrana Transmontana	Kids, male and female	*Longissimus*	5.85	Teixeira et al. [15]
East African Tanzania indigenous goats	1.5–2 years, male and female	*Longissimus thoracis et lumborum*	5.9	Shija et al. [88]
Sarda goats	Kids, male and female	*Longissimus dorsi*	5.9	Vacca et al. [89]
Saanen	Male kids (80 and 120 days)	*Longissimus dorsi*	5.9–6.1	Yalcintan et al. [99]
Guanzhong dairy breed	6–10 m	*Longissimus thoracis*	6.1	Ding et al. [86]

**Table 8:** Reported shear force values of goat meat chilled for 24 h and cooked at an internal temperature ranging from 70–85 °C.

Breed	Age and Sex	Muscle	Cooking Method	Shear Force (kg/cm^2^)	Source
Guanzhong dairy breed and its crosses	6–10 m, unspecified sex	LT	Water bath to 70 °C internal temperature	4.9–6.3	Ding et al. [86]
Serrana Transmontana	Male and female kids	*longissimus*	Water bath to 70 °C internal temperature	6.95	Teixeira et al. [15]
Australian feral	Kids to adult entire and castrated male	LTL	Water bath at 85 °C for 45 min	7.48	Werdi Pratiwi et al. [116]
Omani goats	One year male	LD	Water bath 70 °C for 90 min	7.2–7.7	Kadim et al. [97]
South African indigenous goats	Castrate and female, young to adult	SM	roasted to 72 °C internal temperature	7.6	Simela et al. [94]
Crossbreeds	Male and female kids	LTL	Water bath at 70 to 75 °C internal temperature	7.8	Dhanda et al. [21]
	Male and female kids	GB	Water bath at 70 to 75 °C internal temperature	10.2	Dhanda et al. [21]
Boer	Kids	SM	Roasted to 72 °C internal temperature	11.1–14.3	Sheridan et al. [80]

SM: *M. semimembranosus*; BF: *M. biceps femoris*; LD: *M. longissimus dorsi. Gluteobiceps* (GB), *longissimus thoracis et lumborum* (LTL), *longissimus thoracis* (LT), *quadriceps femoris* (QF), and *gluteobiceps* (GB). Kids: 8–20 kg live weight; young: 1–2 years old goat; adult 2 to 6 years old.

## Data Availability

No new data were created or analyzed in this study. Data sharing is not applicable to this article.
